# Effects of Socio-Demographic, Personality and Medical Factors on Quality of Life of Postmenopausal Women

**DOI:** 10.3390/ijerph110706692

**Published:** 2014-06-26

**Authors:** Sylwia Wieder-Huszla, Małgorzata Szkup, Anna Jurczak, Agnieszka Samochowiec, Jerzy Samochowiec, Marzanna Stanisławska, Iwona Rotter, Beata Karakiewicz, Elżbieta Grochans

**Affiliations:** 1 Department of Nursing, Pomeranian Medical University in Szczecin, 48 Żołnierska St., 71-210 Szczecin, Poland; E-Mails: sylwiahuszla@op.pl (S.W.-H.); m.szkup@onet.eu (M.S.); jurczaka@op.pl (A.J.); s-kaktus1@wp.pl (M.S.); 2 Department of Clinical Psychology, Institute of Psychology, University of Szczecin, 18 Szwoleżerów St., 71-79 Krakowska, Poland; E-Mail: samoaga@tlen.pl; 3 Department of Psychiatry, Pomeranian Medical University in Szczecin, 26 Broniewskiego St., 71-460 Szczecin, Poland; E-Mail: samoj@sci.pam.szczecin.pl; 4 Department of Rehabilitation Medicine, Szczecin University in Szczecin, 31 Grudziądzka St., 70-103 Szczecin, Poland; E-Mail: iwrot@wp.pl; 5 Public Health Department, Pomeranian Medical University in Szczecin, 48 Żołnierska St., 71-210 Szczecin, Poland; E-Mail: karabea@sci.pam.szczecin.pl

**Keywords:** postmenopausal women, quality of life, personality, climacteric symptoms, age

## Abstract

Numerous studies show that changes occurring in a woman’s organism during menopause may lower her quality of life. This study involved 630 healthy postmenopausal women from Poland. Its purpose was to assess their quality of life in relation to socio-demographic variables, medical data and personality profiles. The authors used the Short Form Health Survey (SF-36) to assess quality of life, the NEO-Five Factor Inventory to measure personality traits, and the Blatt-Kupperman Menopausal Index to estimate severity of climacteric symptoms. The study demonstrated significant relationships between quality of life and variables such as: age, education, employment status, and the use of menopausal hormone therapy. An analysis of personality traits revealed correlations between the openness to experience scores and the quality of life within physical functioning, vitality, and mental health. Neuroticism, agreeableness and extroversion significantly correlated with all quality of life domains. Conclusions: (1) Age, education and employment status have significant effects on the selected quality of life domains after menopause. (2) Quality of life within the general health domain was assessed lower by MHT-users (Menopausal hormone theraphy (MHT)). (3) Health-related quality of life is also influenced by personality traits, which are relatively stable throughout life.

## 1. Introduction

Menopause is a biological event associated with a complete cessation of a woman’s reproductive ability as a consequence of the physiological ageing process. In Poland, women usually reach menopause between the ages of 48–52 years [1], while in the World the range is between the ages of 40–58, with the average age being 51 years [2]. Factors which contribute to early menopause are mainly nicotinism, malnutrition, elimination diets [3,4] as well as race, socio-economic status, domicile, number of childbirths, genetic factors [3,5,6,7,8,9], the age of the first menstruation, the use of contraceptives, and alcohol consumption [1,2,10].

Women’s growing awareness causes that they treat menopause as the end of a certain stage followed by a new one. For many of them middle age is the most harmonious time of life [11,12]. Nevertheless, despite many new opportunities, it also involves menopause-related complaints and disorders*.* Hormone changes observed in this period are manifested by diverse symptoms, among them: changes in menstrual cycle, vasomotor and psycho-emotional disorders, urogenital dysfunctions, cardiovascular diseases, lipid disorders, osteoporosis, type 2 diabetes mellitus, breast and uterine cancers, and mental disorders [10,13,14]. Numerous studies show that changes in an organism and related complaints may lower the quality of life after menopause [15], especially in the psychological, physical and sexual spheres [16,17]. A decline in the quality of live at this age may be associated with factors such as: vasomotor disorders [18], sleep problems [19], limitations in psycho-social functioning [20], chronic disorders such as arthritis and migraine [21], osteoporosis [22,23], hypertension, degenerative arthritis, idiopathic chronic back pain, varicose veins, hyperlipidemia, thyroid diseases, diabetes [24] and obesity [25].

One more issue which may influence quality of life is personality, which is a relatively stable element defining one’s tendency to react and interpret situations in a particular manner despite changing circumstances. According to available reports personality plays a role in health-related quality of life of both healthy and ill individuals [26]. This relationship was especially evident in patients suffering from mental diseases, such as affective disorders (including depression), schizophrenia and schizoaffective disorder [27,28]. At present, personality analysis is mainly based on a five-factor model, covering such aspects as neuroticism (tendency to experience negative emotions); extroversion (engagement with the external world, positive and optimistic attitude, sociability); openness to experience (intellectual curiousity, appreciation of art, sensitivity to beauty); agreeableness (altruistic nature, people-oriented attitude); conscientiousness (organizing skills; ability to develop motivation and perseverance in striving after goals ). The above mentioned personality types have solid theoretical foundations and empirical applications [29]. The aim of this study is to assess quality of life after menopause in relation to socio-demographic variables (age, marital status, employment status, education), medical data (the use of MHT, severity of climacteric symptoms) and personality profile.

## 2. Methods and Materials

### 2.1. Subjects

The study involved 630 women from northern Poland who had their last menstrual period at least one year before the study. These women did not abuse alcohol [30], cigarettes, benzodiazepines [31], had not been diagnosed as having endocrinological, cancerous or mental diseases, had undergone neither hysterectomy or oophorectomy. The criteria for inclusion in the study were: a normal cervical smear result, a normal mammography result, no history of thyroid or cancerous diseases, and no psychiatric treatment by the time. The women who met the above criteria were informed about the possibility of taking part in the study by their gynaecologists. Next, they voluntarily reported to the research centre, where they completed questionnaires and had blood samples taken for an analysis. This method guaranteed a 100% return of questionnaires. After examination, all patients received their examination results free of charge. The study was conducted with the consent of the Bioethical Commission of Pomeranian Medical University in Szczecin (permission No. KB-0080/187/09).

### 2.2. Assessments

Quality of life was evaluated with a standardized questionnaire, the Short Form Health Survey (SF*-*36), consisting of 36 questions (items), which measure physical and mental health in relation to eight health aspects, namely: physical functioning (PF); limitations in daily activities due to physical problems (RP); bodily pain (BP); general health (GH); vitality (V); social functioning (SF); role-emotional (RE); mental health (MH). The quality of life score is a sum of the scores obtained in the eight quality of life scales. Participants can obtain 0–100 points for each domain. A higher score corresponds with a higher level of satisfaction with the quality of life. The score of 50 corresponds with the average quality of life, the scores below 50 reflect lower quality of life, and the scores above 50—higher up to excellent quality of life.

An analysis of personality profiles was performed using the Neuroticism-Extroversion-Openness-Five Factor Inventory (NEO-FFI), which consists of five scales measuring: neuroticism, extraversion, openness to experience, agreeableness and conscientiousness [32,33].

The patients used the Blatt-Kupperman Menopausal Index [34] to assess the severity of 11 menopausal symptoms, such as: hot flushes, sweats, insomnia, nervousness, melancholia, vertigo, weakness, arthralgia, headache, palpitations and paraesthesia. Each symptom on the Blatt-Kupperman Menopausal Index was rated from 0 to 3 for no, slight, moderate, and severe complaints. To calculate the Blatt-Kupperman Menopausal Index, the symptoms were weighted as follows: hot flushes (×4), paraesthesia (×2), insomnia (×2), nervousness (×2), and all other symptoms (×1). The highest potential score is thus 51. The scores obtained were interpreted as follows: 0–16 points—no menopausal symptoms, 17–25 points—mild symptoms, 26–30 points—moderate symptoms, and more than 30 points—severe symptoms.

### 2.3. Statistical Analyses

The study group was described in terms of socio-demographic data with the use of a structure ratio. Furthermore, average quality of life scores with standard deviations were given for each of the scales. An analysis of variance (ANOVA) and the Kruskal-Wallis test were employed to detect differences between many samples. We used Pearson’s linear correlation and R-Spearman’s rank correlation coefficient. The analysis results were shown in the tables. Parametric tests were used when assumptions about normal distribution and homogeneity of variance were met. The level of significance was assumed at α = 0.05.

## 3. Results

The average age of the respondents was 57.5 ± 6.4. years Over half of them (51.3%) had secondary education and lived in urban areas with a population of more than 100,000 people (63.81%). A vast majority of the women were married or lived in cohabitation (69.05%); more than a half (56.51%) were employed. Most women did not have climacteric symptoms, only 8.7% had severe symptoms. A vast majority of the surveyed did not use MHT—66.83% (Table 1).

**Table 1:** Group structure according to socio-demographic and medical data of respondents.

Socio-demographic Data	*n*	%
Education		
Primary	19	3.0
Vocational	64	10.2
Secondary	323	51.3
University	222	35.2
No answer	2	0.3
Total	630	100.0
Domicile		
Village	55	8.7
City/town of less than 10,000 people	35	5.6
City/town of 10,000–100,000 people	121	19.2
City/town of more than 100,000 people	402	63.8
No answer	17	2.7
Total	630	100.0
Marital status		
Married	435	69.1
Unmarried	191	30.3
No answer	4	0.6
Total	630	100.0
Employment		
Employed	356	56.5
Unemployed	269	42.7
No answer	5	0.8
Total	630	100.0
Medical data	*n*	%
Use of menopausal hormone therapy		
Yes	205	32.6
No	421	66.8
No answer	4	0.6
Total	630	100.0
Climacteric symptoms according to the Blatt-Kupperman Menopausal Index		
Absent	365	57.9
Mild	152	24.1
Moderate	58	9.3
Severe	55	8.7
Total	630	100.0

The highest quality of life scores were obtained within physical functioning (77.1 ± 19.8), role-emotional (75.6 ± 37.1), and social functioning (71.9 ± 24.8). The lowest scores were noted in general health (50.9 ± 16.3) and bodily pain (55.5 ± 25.3) (Table 2).

**Table 2:** Quality of life of respondents according to the SF-36.

Scale	*n*	χ ± SD	Min–Max
Physical functioning (RF)	623	77.1 ± 19.8	0–100
Limitations in daily activities due to physical problems (RP)	629	63.9 ± 38.4	0–100
Bodily pain (BP)	623	55.5 ± 25.3	0–100
General health (GH)	616	50.9 ± 16.3	5–100
Vitality (V)	622	58.5 ± 19.1	5–100
Social functioning (SF)	623	71.9 ± 24.8	0–100
Role-emotional(RE)	628	75.6 ± 37.1	0–100
Mental health (MH)	621	65.6 ± 17.9	8–100

Note: **χ ±**SD—mean and standard deviation, Min-minimum value, Max-maximum value.

An analysis of personality traits showed that their intensity was at the medium level (Table 3).

**Table 3:** Personality traits of respondents according to NEO-FFI.

Level	Personality Traits									
	Openness to Experience		Neuroticism		Agreeableness		Extraversion		Conscientiousness	
	*n*	%	*n*	%	*n*	%	*n*	%	*n*	%
Low	76	12.1	215	34.2	69	11.0	86	13.7	80	12.7
Medium	270	43.0	269	42.8	340	54.1	315	50.2	382	60.8
High	282	44.9	144	22.9	219	34.9	227	36.1	166	26.4

The quality of life results were divided into three groups depending on a type of variables:

1. Socio-demographic (age, education, employment status, domicile, marital status)
2. Medical (severity of climacteric symptoms, using or not using MHT)
3. Personality

### 3.1. The Quality of Life Analysis in Relation to the Selected Socio-Demographic Data

A correlation analysis indicates a significant relationship between the age of the women and their quality of life within physical functioning, limitations in daily activities due to physical problems, and vitality (*p* < 0.05). Correlation coefficients ranged from −0.156 to 0.171, which means that the correlations were weak. The quality of life in other domains did not correlate with age (*p* > 0.05) (Table 4).

**Table 4:** Analysis of correlations between quality of life according to SF-36 and age of respondents.

Scale	Age	
	*r*	*p*
Physical functioning (PF)	−0.1555	<0.05
Limitations in daily activities due to physical problems (RP)	−0.1088	<0.05
Bodily pain (BP)	−0.0564	>0.05
General health (GH)	0.0227	>0.05
Vitality (V)	0.1707	<0.05
Social functioning (SF)	0.1044	>0.05
Role-emotional(RE)	−0.0212	>0.05
Mental health (MH)	0.0681	>0.05

Note: Pearson’s linear correlation coefficient (*r*), level of significance for *r*.

An analysis of data in relation to education demonstrated statistically significant differences within bodily pain and mental health of postmenopausal women (*p* < 0.05). No statistically significant differences within other scales were observed (*p* > 0.05) (Table 5).

**Table 5:** Quality of life of respondents according to SF-36 with regard to education and domicile.

Scale	χ ± SD	χ ± SD	χ ± SD	χ ± SD	ANOVA Test Value	*p*
	Education					
	Primary	Vocational	Secondary	University		
Physical functioning (PF)	71.1 ± 19.8	68.6 ± 25.0	76.9 ± 20.0	80.2 ± 16.7	2.96 ^KW^	>0.05
Limitations in daily activities due to physical problems (RP)	61.8 ± 42.8	55.5 ± 39.2	63.4 ± 38.0	66.9 ± 38.1	1.51 ^A^	>0.05
Bodily pain (BP)	50.8 ± 23.6	50.3 ± 25.3	52.8 ± 25.3	61.5 ± 24.6	6.55 ^A^	<0.05
General health (GH)	43.8 ± 10.7	51.1 ± 14.1	50.9 ± 15.7	51.4 ± 18.0	1.71 ^KW^	>0.05
Vitality (V)	58.7 ± 17.2	56.6 ± 16.6	57.9 ± 18.7	60.1 ± 20.4	3.03 ^KW^	>0.05
Social functioning (SF)	71.7 ± 24.2	71.0 ± 23.9	71.4 ± 24.8	73.1 ± 23.9	0.22 ^A^	>0.05
Role-emotional(RE)	70.2 ± 41.4	67.2 ± 39.9	76.5 ± 36.9	77.0 ± 36.1	1.39 ^A^	>0.05
Mental health (MH)	65.5 ± 16.2	61.3 ± 18.1	63.9 ± 18.0	69.2 ± 17.3	5.13 ^A^	<0.05
**Scale**	**χ ± SD**	**χ ± SD**	**χ ± SD**	**χ ± SD**	**ANOVA Test Value**	***p***
	**Domicile**				**ANOVA**	
	**Village**	**Town/City of Less Than 10,000 People**	**Town/City of 10,000–100,000 People**	**Town/City of More Than 100,000 People**		
Physical functioning (PF)	71.2 ± 23.9	75.6 ± 19.8	77.1 ± 17.4	78.0 ± 19.7	2.02	>0.05
Limitations in daily activities due to physical problems (RP)	65.0 ± 39.2	66.2 ± 38.9	64.1 ± 39.4	63.3 ± 38.1	0.09	>0.05
Bodily pain (BP)	51.4 ± 24.9	54.9 ± 27.0	56.2 ± 24.3	55.8 ± 25.5	0.53	>0.05
General health (GH)	52.8 ± 18.4	46.7 ± 14.7	49.9 ± 15.3	50.9 ± 16.4	1.11	>0.05
Vitality (V)	59.5 ± 18.9	53.5 ± 17.6	58.3 ± 18.3	58.6 ± 19.4	0.84	>0.05
Social functioning (SF)	70.0 ± 25.1	69.5 ± 26.7	72.3 ± 24.8	72.5 ± 24.5	0.30	>0.05
Role-emotional(RE)	75.8 ± 37.1	77.5 ± 36.4	78.3 ± 37.6	74.4 ± 37.1	0.39	>0.05
Mental health (MH)	65.8 ± 19.1	61.4 ± 17.8	64.9 ± 17.4	65.9 ± 17.9	0.75	>0.05

Note: χ **±** SD—mean and standard deviation, ^A^ analysis of variance (ANOVA), ^KW^ non-parametric equivalent of ANOVA: Kruskal-Wallis test, *p* level of significance.

A detailed analysis demonstrated that women with secondary education had significantly lower quality of life within bodily pain and mental health than women with higher education (*p* < 0.05).

Statistically significant differences in the quality of life of the postmenopausal women in relation to their domicile were not observed in any domain (*p* > 0.05) (Table 5). An analysis did not confirm that the marital status of the surveyed women significantly influenced their quality of life in any domain (*p* > 0.05) (Table 6).

Employed women had higher quality of life within physical functioning, limitations in daily activities due to physical problems, bodily pain, and role emotional (*p* < 0.05). In other scales there were no differences in the quality of life between employed and unemployed women (*p* > 0.05) (Table 6).

### 3.2. A Quality of Life Analysis in Relation to the Selected Medical Data

There were statistically significant differences in general health between users and non-users of MHT. MHT-users had significantly lower quality of life within general health (*p* < 0.05). No statistically significant differences were observed in other domains (*p* > 0.05) (Table 6).

An analysis of data demonstrated statistically significant correlations between the openness to experience scores according to the NEO-FFI and the quality of life within physical functioning, vitality, and mental health (*p* < 0.05). The relationship between these variables was weak, and the correlation almost insignificant.

Neuroticism correlated significantly with all quality of life domains (*p* < 0.05). A moderate correlation was observed (*R* = −0.54) within mental health. There was a distinct but small relationship between a neurotic personality and the quality of life within other domains. In all cases negative correlations were noted, which means that higher quality of life scores were accompanied by low levels of neuroticism. This shows that women who were relaxed, free of tension and fears, and able to cope with stressful situations had higher quality of life.

**Table 6:** Quality of life of respondents according to SF-36 with regard to marital status, employment and the use of MHT.

Scale	χ ± SD	χ ± SD	ANOVA Test Value	*p*
	Married *n* = 435	Unmarried *n* = 191		
Physical functioning (PF)	77.5 ± 19.3	76.1 ± 20.7	0.83 ^T^	>0.05
Limitations in daily activities due to physical problems (RP)	64.8 ± 38.1	61.7 ± 39.0	0.93 ^T^	>0.05
Bodily pain (BP)	55.4 ± 24.7	55.8 ± 26.8	−0.19 ^T^	>0.05
General health (GH)	51.2 ± 16.2	50.1 ± 16.4	0.78 ^T^	>0.05
Vitality (V)	58.6 ± 19.3	58.5 ± 18.5	0.07 ^T^	>0.05
Social functioning (SF)	73.2 ± 24.6	69.3 ± 25.1	1.82 ^T^	>0.05
Role-emotional(RE)	76.9 ± 36.2	72.4 ± 38.9	1.34 ^MW^	>0.05
Mental health (MH)	66.2 ± 17.7	64.2 ± 18.4	1.28 ^T^	>0.05
	Employed *n* = 356	Unemployed *n* = 269		
Physical functioning (PF)	81.2 ± 17.1	72.0 ± 21.6	−5.52 ^MW^	<0.05
Limitations in daily activities due to physical problems (RP)	74.2 ± 34.4	50.2 ± 39.2	−7.60 ^MW^	<0.05
Bodily pain (BP)	59.7 ± 25.8	50.1 ± 23.7	−4.75 ^T^	<0.05
General health (GH)	51.5 ± 15.6	50.1 ± 17.2	−1.04 ^T^	>0.05
Vitality (V)	58.9 ± 18.7	58.2 ± 19.7	−0.48 ^T^	>0.05
Social functioning (SF)	72.7 ± 24.8	71.2 ± 24.8	−0.78 ^T^	>0.05
Role-emotional(RE)	80.7 ± 34.4	68.9 ± 39.6	−4.10 ^MW^	<0.05
Mental health (MH)	66.1 ± 16.6	65.0 ± 19.5	−0.53 ^T^	>0.05
	MHT—yes *n* = 205	MHT—no *n* = 421		
Physical functioning (PF)	78.8 ± 18.4	76.2 ± 20.3	1.22 ^MW^	>0.05
Limitations in daily activities due to physical problems (RP)	67.6 ± 37.8	61.9 ± 38.6	1.75 ^T^	>0.05
Bodily pain (BP)	54.7 ± 25.6	55.9 ± 25.3	−0.56 ^T^	>0.05
General health (GH)	48.8 ± 14.7	51.8 ± 16.9	−2.12 ^T^	<0.05
Vitality (V)	59.0 ± 17.9	58.3 ± 19.6	0.42 ^T^	>0.05
Social functioning (SF)	74.0 ± 24.7	71.0 ± 24.9	1.38 ^T^	>0.05
Role-emotional(RE)	78.6 ± 34.1	74.1 ± 38.4	1.01 ^MW^	>0.05
Mental health (MH)	66.0 ± 17.3	65.4 ± 18.6	0.05 ^MW^	>0.05

Note: χ **±** SD—mean and standard deviation, ^A^ analysis of variance (ANOVA), ^KW^ non-parametric equivalent of ANOVA: Kruskal-Wallis test, *p* level of significance.

Agreeableness statistically significantly correlated with quality of life in nearly all domains (*p* < 0.05). The only exception was general health (*p* > 0.05)—in all other cases there was a weak relationship. This proves that women who show positive friendly attitudes toward other people, are altruistic, emphatic and eager to co-operate with others have higher quality of life.

Extraversion correlated significantly with all quality of life domains (*p* < 0.05). This was reflected by higher quality of life scores obtained by women with higher self-energy, a sociable and friendly but assertive disposition, women who were active, searched for new experiences and were open to positive emotions.

Almost all quality of life domains (aside from general health) statistically significantly correlated with conscientiousness (*p* < 0.05). There were obvious but weak correlations with role emotional and mental health (*R* = 0.21 and 0.24 respectively). In other scales the relationship with conscientiousness was weak (Table 7).

**Table 7:** Correlations between quality of life according to SF-36 and personality traits according to NEO-FFI.

Scale	*n*	NEO-FFI									
		Openness to Experience		Neuroticism		Agreeableness		Extraversion		Conscientiousness	
		*R*	*p*	*R*	*p*	*R*	*p*	*R*	*p*	*R*	*p*
Physical functioning (PF)	623	0.11	<0.05	−0.22	<0.05	0.15	<0.05	0.23	<0.05	0.18	<0.05
Limitations in daily activities due to physical problems (RP)	627	0.01	>0.05	−0.26	<0.05	0.17	<0.05	0.23	<0.05	0.19	<0.05
Bodily pain (BP)	623	0.06	>0.05	−0.30	<0.05	0.13	<0.05	0.21	<0.05	0.08	<0.05
General health (GH)	616	0.06	>0.05	−0.27	<0.05	0.07	>0.05	0.21	<0.05	0.06	>0.05
Vitality (V)	622	0.10	<0.05	−0.40	<0.05	0.10	<0.05	0.40	<0.05	0.17	<0.05
Social functioning (SF)	623	0.08	>0.05	−0.38	<0.05	0.21	<0.05	0.36	<0.05	0.19	<0.05
Role-emotional(RE)	626	0.07	>0.05	−0.37	<0.05	0.12	<0.05	0.28	<0.05	0.21	<0.05
Mental health (MH)	621	0.12	<0.05	−0.54	<0.05	0.20	<0.05	0.37	<0.05	0.24	<0.05

Note: R-Spearman’s rank correlation coefficient, *p* level of significance for *R*.

An analysis of data revealed that all quality of life scales correlated negatively with the Blatt-Kupperman Menopausal Index (*p* < 0.05), which means that the quality of life decreased with an increasing severity of climacteric symptoms.

**Table 8:** Correlations between quality of life according to the SF-36 and severity of climacteric symptoms according to the Blatt-Kupperman Menopausal Index.

Scale	*n*	*R*	*p*
Physical functioning (PF)	623	−0.327163	<0.05
Limitations in daily activities due to physical problems (RP)	629	−0.323632	<0.05
Bodily pain (BP)	623	−0.464898	<0.05
General health (GH)	616	−0.301235	<0.05
Vitality (V)	622	−0.375406	<0.05
Social functioning (SF)	623	−0.409584	<0.05
Role-emotional(RE)	628	−0.358003	<0.05
Mental health (MH)	621	−0.357593	<0.05

Note: R-Spearman’s rank correlation coefficient, *p* level of significance for *R*.

In the majority of cases there were obvious but insignificant relationships with personality traits. Only bodily pain and social functioning correlated moderately with climacteric symptoms (correlation coefficients were −0.46 and −0.41 respectively) (Table 8).

## 4. Discussion

The quality of life after menopause may depend on many issues, including age. American studies show that an advanced age is a risk factor of a lower quality of life [35]. Similar conclusions were also drawn by Calvo-Pérez and Campillo-Artero, who analysed a group of women from Mallorca (Spain) [36]. Furthermore, studies conducted in Poland with the use of the SF-36 questionnaire demonstrated negative correlations between age and self-reported physical health [37,38,39]. Age negatively affected the quality of life of Chinese women, especially in the context of vasomotor and sexual disorders [40]. In German women, on the other hand, age correlated with cardio-pulmonary symptoms and sensory impairment [24]. An Iranian analysis proved that age had a profound impact on quality of life, with emphasis put on its contribution to the occurrence of vasomotor disorders [41]. The outcomes of the study carried out in Ecuador suggest that quality of life is linked to age and age-related problems, such as: abdominal obesity, hyperglycaemia and hypertension [17]. In the study described in this article, the women’s age significantly correlated with their quality of life within physical functioning and limitations in daily activities due to physical problems.

According to Trafalska *et al.* education, employment status, and domicile have effects on quality of life within physical functioning and mental health [42]. An impact of education on these two aspects of health was also confirmed by Szczepańska’s team [43]. In Poland, the highest scores for quality of life within physical functioning and mental health were achieved by well-educated employed women living in large urban areas [44]. The research conducted in the US suggests that a low level of education and low income are risk factors of a decline in quality of life [35]. The relationship between a level of education and life satisfaction was also observed among postmenopausal women from Mallorca [36]. The life satisfaction of Turkish women correlated with their education, income level, employment status, regular sport exercises, chronic diseases, and social support from family and friends [45]. Furthermore, in the study presented in this article, education had significant effects on quality of life, especially within bodily pain and mental health. Women with higher education assessed their quality of life in these domains considerably better. However, significant correlations between quality of life and such variables as domicile and marital status were not demonstrated.

Numerous studies show that an element which is particularly important for postmenopausal women is an employment status. Its influence on quality of life was proved by Trafalska *et al.* [42], Marcinkowski *et al.* [46], Kowalska *et al.* [47], Karczewski *et al.* [48], Bal *et al.* [45], Zack *et al.* [35]. Iranian studies suggest that being employed and having children decrease a risk of a decline in quality of life within psycho-social functioning [41]. A survey of American women revealed that employment status aside, quality of life after menopause highly depends also on such factors as: age, smoking, level of education, sport exercises, and BMI [18]. The authors of this study observed correlations between employment status and quality of life of postmenopausal women. Employed women had higher quality of life within physical functioning, limitations in daily activities due to physical problems, bodily pain, and role emotional.

Although there are many contradictory opinions about the efficiency and safety of this form of treatment, MHT is often regarded as the best way to eliminate consequences of the menopausal period. According to Taylor and Manson, MHT reduces hot flushes and vasomotor symptoms, alleviates sleep problems, and improves sexual functioning [49]. Even if they experience troublesome symptoms, women are often afraid of starting the therapy, since numerous reports indicate its side-effects, such as: putting on weight, androgenisation and neoplastic diseases. Some researchers emphasize favourable influence of MHT on the reduction of negative consequences of sex hormone deficiency, claiming that the therapy tailored to individual needs does not induce obesity. A number of women taking hormone therapy is not large [38,50], which was confirmed by the presented findings (in the study group, merely 32.54% of women used MHT). According to some authors, MHT improves quality of life within physical functioning [39,42,46]. This, however, does not correspond with the results obtained in the study presented here, since women who did not use MHT assessed their health better than those who did. Similar results were reported by Marcinkowska *et al.* [51]. In the study of Henrykowska, quality of life within mental health was better evaluated by MHT non-users [52]. The findings obtained by Żołnierczuk-Kieliszek did not provide unambiguous evidence for a positive influence of MHT on the quality of women’s lives [53]. In the research of Avis *et al.* present and past MHT-users considered their quality of life to be low in all domains more often than those who had never used hormone therapy [21]. Similar results were obtained by Mishra *et al.* who noticed that MHT contributed to worse functioning in all SF-36 domains, except for role emotional [54]. These findings seem to be opposite to a general impression that women taking MHT are healthier. It is possible that this assumption is based on more objective health evaluation than subjective opinions of respondents [55].

The analysis results suggest that there is a relationship between personality traits defined according to the NEO-FFI and different aspects of health-related quality of life, measured with the SF-36 questionnaire. The study of Schimmack *et al.* proved that neuroticism and extraversion are related to quality of life and subjective health assessment, both in healthy people and those with somatic disorders [56]. In the study of van Straten *et al.* individuals with high agreeableness scores had higher quality of life in social and physical spheres, but not in emotional one. It may result from the fact that such people usually have more friends, are more relaxed and do not attach too much weight to pain and physical discomfort [26]. The research conducted in Turkey shows that higher levels of introversion and neuroticism are accompanied by lower quality of life after menopause [45]. This study, on the other hand, proves that higher levels of agreeableness are connected to higher quality of life in all domains, except for general health.

Extraversion is one more element having positive effects on quality of life. It is assumed that people who are involved in interaction with others, are less concentrated on their own problems and, consequently, better assess their own functioning [26]. The results presented in this article show that extraversion has positive effects on all aspects of quality of life.

Many personality studies provide evidence that individuals with mental disorders obtain results different from those achieved by their healthy counterparts [57]. It has been proved that especially high neuroticism is associated with changes in mental health [58]. The reasons for this relationship have not been fully understood so far [59]. Nevertheless, it was noticed that irrespective of whether highly neurotic people suffer from mental disorders or not, neuroticism has a profound impact on their health-related quality of life. Negative effects of severe neuroticism on quality of life were observed in all aspects analysed in this study.

The research conducted among 8,811 postmenopausal women in the US showed that women suffering from menopausal symptoms have significantly lower quality of life, more often lose or resign from their jobs, and need more support from health care institutions. It was also found that climacteric symptoms may be an important factor affecting the efficiency of middle-aged women, both in the aspect of their personal functioning and financial standing [60]. The study performed on a group of 4437 Taiwanese women proved that age, religion, smoking, sport exercises, and diseases were independent contributors to the expression of menopausal symptoms. The authors noted that the occurrence of these symptoms entailed a decline in health-related quality of life within physical functioning and mental health [61]. Kumari *et al.* carried out a study on 2489 women to assess their quality of life with special attention paid to the effects of menopausal symptoms on particular SF-36 domains. It was observed that women with vasomotor disorders and depression had significantly lower quality of life [62]. Furthermore, the outcomes of an analysis performed by Avis *et al.* confirm that climacteric symptoms influence quality of life. Vaginal dryness, urinary incontinence, sleep disorders and depression were proved to play a role in the decline in quality of life in all SF-36 domains [21]. This study also shows the significance of climacteric symptoms for the functioning of postmenopausal women—menopausal symptoms lowered quality of life in all domains. Quality of life studies performed on general population show that it functions better in all quality of life domains comparing to postmenopausal women (for ex. according to the research conducted in Brazil, general population has a 8.2% higher quality of life than postmenopausal women) (PF 82.45 *vs.* 77.1, RP 74.73 *vs.* 63.9, BP 67.53 *vs.* 55.5, GH 71.10 *vs.* 50.9, V 66.85 *vs.* 58.5, SF 78.30 *vs.* 71.9, RE 70.02 *vs.* 75.6, MH 73.82 *vs.* 65.6) [63].

The research described in this article has several valuable aspects. First of all, the study was performed on a large group of women. Furthermore, all participants were surveyed using standardised research tools, and met clearly defined inclusion criteria. A limitation of the study is the fact that not all socio-demographic data, potentially having an impact on health-related quality of life, were taken into consideration, and this is the aspect which requires further analyses.

## 5. Conclusions

(1) Socio-demographic data having significant effects on the selected quality of life domains after menopause are: age, education and employment status.
(2) Quality of life within the general health domain was assessed lower by MHT-users.
(3) Health-related quality of life is not only influenced by pathological symptoms, present life situation, and socio-demographic variables, but also personality traits, which are relatively stable throughout life.
